# A Nanofluidic Biosensor Based on Nanoreplica Molding Photonic Crystal

**DOI:** 10.1186/s11671-016-1644-x

**Published:** 2016-09-23

**Authors:** Wang Peng, Youping Chen, Wu Ai, Dailin Zhang

**Affiliations:** 1 School of Mechanical Science and Engineering, Huazhong University of Science and Technology, Wuhan, 430074 China; 2 Department of Electrical and Computer Engineering, University of Illinois at Urbana-Champaign, Urbana, IL 61801 USA; 3 Micro and Nanotechnology Laboratory, University of Illinois at Urbana-Champaign, Urbana, IL 61801 USA

**Keywords:** Photonic crystals, Nanochannels, Optofluidic biosensor, Nanoreplica molding

## Abstract

A nanofluidic biosensor based on nanoreplica molding photonic crystal (PC) was proposed. UV epoxy PC was fabricated by nanoreplica molding on a master PC wafer. The nanochannels were sealed between the gratings on the PC surface and a taped layer. The resonance wavelength of PC-based nanofluidic biosensor was used for testing the sealing effect. According to the peak wavelength value of the sensor, an initial label-free experiment was realized with R6g as the analyte. When the PC-based biosensor was illuminated by a monochromatic light source with a specific angle, the resonance wavelength of the sensor will match with the light source and amplified the electromagnetic field. The amplified electromagnetic field was used to enhance the fluorescence excitation result. The enhancement effect was used for enhancing fluorescence excitation and emission when matched with the resonance condition. Alexa Fluor 635 was used as the target dye excited by 637-nm laser source on a configured photonic crystal enhanced fluorescence (PCEF) setup, and an initial PCEF enhancement factor was obtained.

**Electronic supplementary material:**

The online version of this article (doi:10.1186/s11671-016-1644-x) contains supplementary material, which is available to authorized users.

## Background

Biosensors, which are used for collecting information from interaction between biomolecules and related sensor environment, have been researched extensively in healthcare, biomedical, and life science area [1–8]. Optical biosensors with intrinsically characteristics of label-free, high signal-to-noise ratio, and easy to integrate are even indispensable in low-concentration detection experiments [9–13]. However, traditional mass transport optical biosensors are very difficult for the analytes to attach to the sensing surface through convection flow and diffusion [14, 15]. Nanochannel has a very high surface-to-volume ratio, which makes it much easier for analyte molecules to bump into the internal surfaces, where it can be captured by recognition molecules such as antibody [16–20]. Especially for low-concentration analytes, the time required for analytes to touch the sensing surface can take many hours with normal-size channels by diffusion, which is an important limitation to the performance of surface biosensors. Therefore, due to the large surface-to-volume ratio, nanochannels have a very short analyte diffusive time, which is an important characteristic for biosensor application [21–24].

To date, there are many nanochannel-based biosensors that have been fabricated [25–27]. A high-performance nanoplasmonic-nanofluidic sensor fabricated with lift-off and e-beam lithography techniques has been sculptured in [28]. Similarly, a flow-through nanohole array based on surface plasmons has been demonstrated in [29]. However, the cost of these fabrication processes is very high, and it is difficult to preserve the suspending which is fragile while liquid flow through nanochannels. Targeted at these problems, a robust optofluidic Fabry-Pѐrot cavity sensor with micro/nanochannels has been designed in [30], but the operation wavelength is fixed due to the structure of the cavity, which restricts its further applications. Innovative approaches, which can make the nanochannel-based biosensors easy to fabricate, low cost, and suitable to mass production, are of key importance.

In this paper, a photonic crystal (PC)-based nanochannel biosensor was proposed and fabricated with nanoreplica-molded PC as the substrate and sealed with a tape layer. Since the initial work of Yablonovitch [31], PC-based biosensors have been demonstrated in various areas [32, 33]. As the sensing area of PC-based biosensor is located around the periodic grating surface, the sensing range is very limited due to the influence of evanescent filed [34, 35]. Furthermore, since the analyte transport is diffusive, most of the PC-based bulk sensors cannot be effectively used for biomolecule or protein detection, which need to attach the analytes to the PC grating surface [36, 37]. Therefore, the innovation of PC-based nanochannel biosensor is drastically needed [38]. Also, as the lowest concentration detection is generally performed with fluorescence labels rather than label-free detection, the photonic crystal-enhanced fluorescence excitation is important to improve the limit of detection.

Unlike the conventional PC fabrication approaches where e-beam lithography process is used [39], the PC in this sensor is made by nanoreplica molding with a master wafer as PC mold and UV curable polymer as the replicating material [40]. The molded polymer with grating structure is used as substrate, and a thin layer of TiO_2_ is deposited on its surface as the guided layer. By tuning the thickness of the coated TiO_2_, the resonance wavelength can be tuned in a certain range for various fluorescence enhancement-based applications.

## Method

**Fig. 1:**
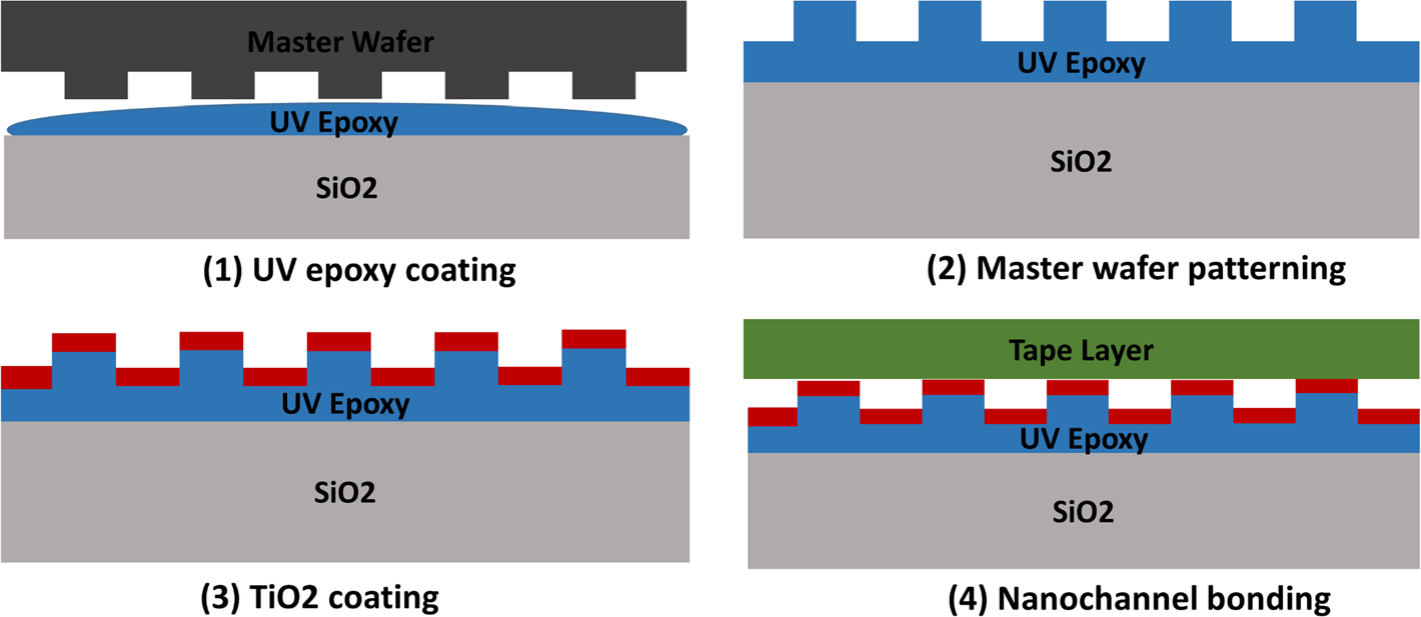
Fabrication process of PC-based nanofluidic biosensor

The fabrication process of nanofluidic chip includes four steps, which are (1) fabrication of master wafer, (2) nanoreplica molding of photonic crystal gratings on the glass based UV curable epoxy, (3) depositing high reflective material TiO2, and (4) direct bonding between PC and laser cutting tape layer, as shown in Fig 1.

**Fig. 2:**
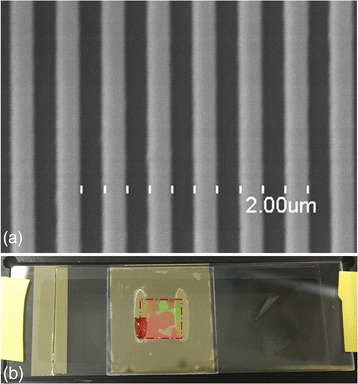
**a** SEM image of PC gratings. **b** Sample of PC-based nanofluidic biosensor, *red dashed box* represents PC grating area

A SiO2 master wafer with patterned area of 8.9mm × 8.9 mm, 400 nm pitch, and 120 nm grating was fabricated with UV lithography and reactive ion etching. The nanoreplica molding process is as follows: a coverslip glass from NEXTERION (75 mm × 25 mm × 1 mm) had been selected as carry substrate, and a Thermal Scientific cover glass (70 mm × 22 mm × 0.17 mm) had been used as molding substrate. Both slides were cleaned by acetone, IPA, DI water, and IPA in sequence, then descum in a Diener O2 plasma (500 W, 3 min). The 0.17-mm-thickness cover glass was sent into a Head Way spinner for coating. Firstly, 10 drops of HMDS were dropped by pipette as adhesive layer, with a spin speed of 3000 rpm for 30 s. Then, 7 drops of Tran Spin HE-0600 were used as nanoimprint material on top of HDMS with 3000 rpm for 30 s. On the pattern transfer step, the SiO2 master wafer was mounted on the surface of a heater (60 °C). Two drops of ZPUA from Gelest Company was dropped on the patterned area of master wafer by a syringe, and it will automatically spread among the surface at 60 °C. Then, the 0.17-mm cover slide was attached on the top of the master wafer and cured with a UV lamp. On the peel off step, NOA 601 adhesive was dropped on the backside of the coated cover slide, and then the 1-mm-thick coverslip was attached on backside of the patterned slide with adhesive. After the NOA 601 cured under UV lamp, the nanoreplica molding pattern can be peeled off from the master wafer. Then, a 70-nm TiO2 was coated on the grating side of the glass as high refractive index layer. The machine used for TiO2 coating is Lesker PVD 75, with 300-W power in vacuum condition for 25 min. As shown in Fig. 2a, the SEM image was taken from Hitachi S-4800 field emission scanning electron microscope, and the gratings of the nanoreplica molding PC structure were fabricated uniformly.

The upper layer of the nanofluidic channel is clear polyolefin film (9795R) with 3-M acrylate adhesive on one side. The inlet and outlet patterns on the upper layer were designed by Inkscape and cut by laser cutting machine Epilog. The upper adhesive layer was attached on the surface of the nanoreplica molding photonic crystal with N2 gas gun. The profile of the nanoreplica molding PC-based biosensor is shown in Fig. 2b. Then, a transmission optical setup was used to obtain the transmission spectrum of the PC-based nanofluidic biosensors. The transmission optical setup was configured with white light source, polarizer, and OSA spectrometer.

### Biosensor Design

Optical biosensors is measuring according to the light principles and characteristics, such as light intensity or/and wavelength during the transduction process. The concept of PC is a periodic grating structure with a high refractive index material as guided layer and surrounded by two relatively lower refractive index layers. When illuminated by a collimated broadband light source, a specific wavelength light will resonate with the PC gratings and reflect back while the remaining wavelength range light passes through the PC structure. When there are analytes on the surface of the PC structure, the resonance wavelength will be modulated and resulting in a resonance wavelength red shift. By measuring the shift distance of the resonance wavelength, the related analyte concentration information can be obtained.

**Fig. 3:**
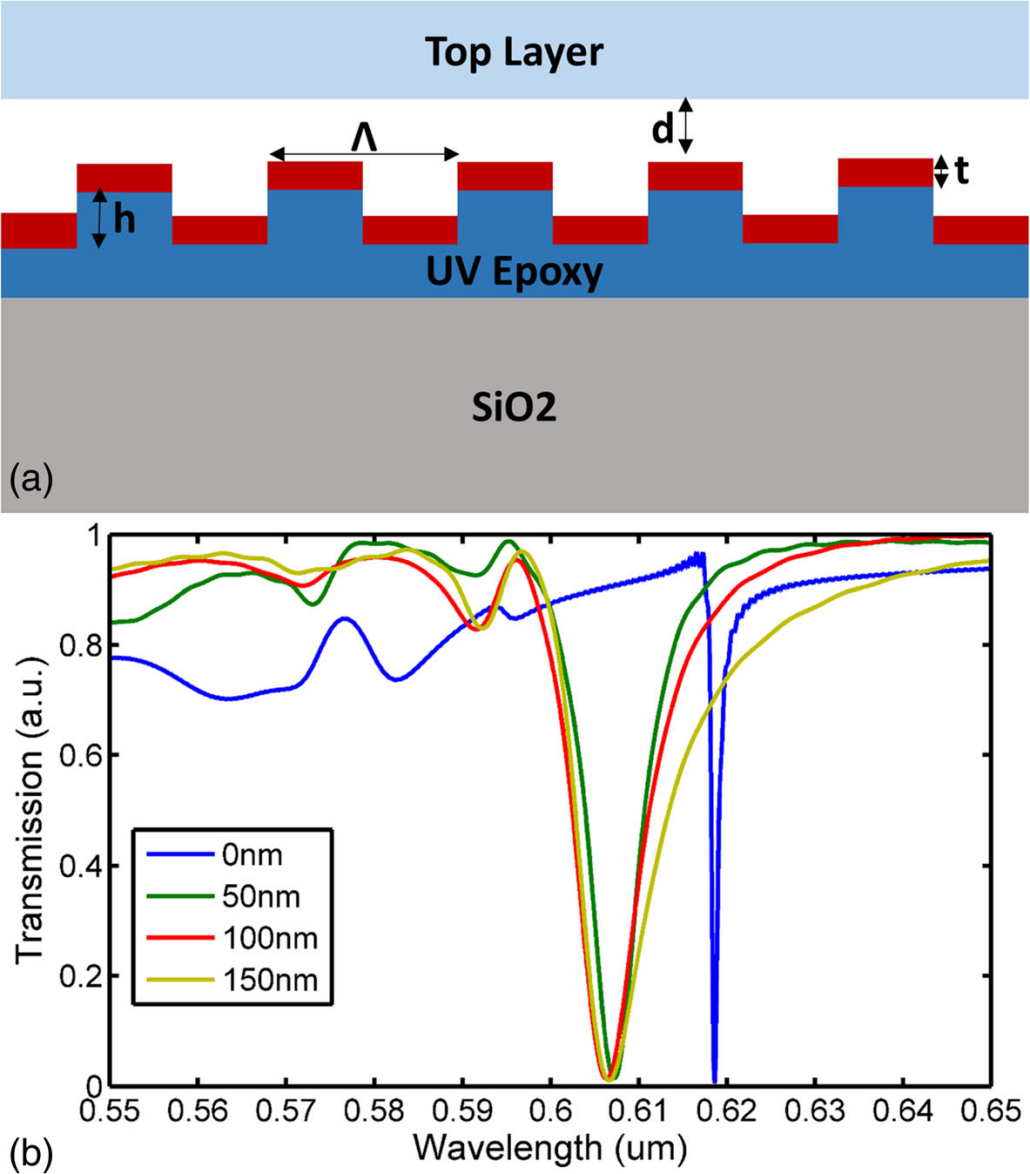
**a** PC-based nanofluidic sensor structure: grating pitch *Λ*, gap distance *d*, TiO2 depth *t*, master wafer grating depth *h*. **b** Transmission spectrum of nanofluidic sensor with various gap distances

The nanofluidic sensor proposed is sealed between a molded PC substrate and a top layer. The sealed gratings can be used as nanofluidic channels according to the proposal. Thus, PCs are commonly used as optical sensors, which have a various applications in biomedical, life science, and healthcare areas. The proposed PC based nanofluidic structure, as shown in Fig. 3a, has a period *Λ* = 400 nm, fillfactor ff = 0.5, UV curable polymer grating depth *h* = 120 nm, and coated TiO2 depth *t* = 70 nm. The potential gap distance between top layer and PC surface is *d*. When the PC and top layer is bonded well with each other, the gap distance *d* is 0. The refractive index of TiO2 is 2.5, UV curable polymer 1.45, and upper covered tape 1.45 [41].

### Bonding Effect Simulation

When bonding result between the PC and top layer is not good enough, there will be a gap distance *d* that will exist, as shown in Fig. 3a. As the peak wavelength value of PC is the redistribution of the electromagnetic field in and near the periodic high refractive index layer, the top sealed layer will have little impact on the redistribution of the near field if it is far away from the top of the TiO2 grating surface. In order to test the influence of gap distance to the final result of biosensor, a gap distance-related simulation was realized. On the simulation stage, FDTD from Lumerical was used as the electrical field and reflection/transmission spectrum simulation tool. When there is a collimated and polarized white light source propagates into the proposed nanofluidic structure at normal incidence, a specified wavelength light will be coupled into the photonic crystal gratings as resonance wavelength and reflected back, while all the remained wavelength range light will pass through the photonic crystal structure. If a detector is used to capture the reflected back light with a reflection setup, a peak resonance wavelength will be shown on the spectrum. If the detector is used to capture the transmitted light with a transmission setup, a dip will be shown on the spectrum. The dip point can also be regarded as the resonance wavelength value, and it is the same value as the peak resonance wavelength. As for the proposed photonic crystal-based nanofluidic sensor, the transmission setup was chosen for the experiments since all the materials have transparency and the light absorption influence can be ignored.

During the simulation, the light source had been set as TM polarized, plane wave (wavelength range 400–700 nm), and incident angle 0°. A virtual detector was plotted on the transmission side of the sensor structure. All the other parameters of the proposed sensor was the same as the fabricated one, and the only tunable parameter was the gap distance d between PC and sealed layer. When the gap distance d was various around 0, 50, 100, and 150 nm in sequence, the related transmission spectrums were calculated by FDTD in sequence. With these transmission spectrum results, the simulation results are shown in Fig. 3b: when PC and sealed layer were totally bonded (gap distance d = 0 nm), the peak wavelength value (PWV) was located at 618.6 nm; when gap distance *d* = 50 nm, the PWV is 607.3 nm; and when the gap distance increases to 100 or 150 nm, the PWV remained on 606.2 nm. From the PWV shift results on Fig. 3b, it can be indicated that when the distance is larger than 50 nm, the upper layer will have almost no impact on the redistribution of the PC EM near field, and also, the PWV would not shift significantly. This phenomenon can be used as a direct method to test the bonding result between the PC and upper cover layer. If the bonding effect is not good enough, the PWV of the bonded sensor will not shift when detected from a transmission optical setup. Otherwise, a significant shift of the PWV represents a solid bonding result.

### Flow Ability Simulation

**Fig. 4:**
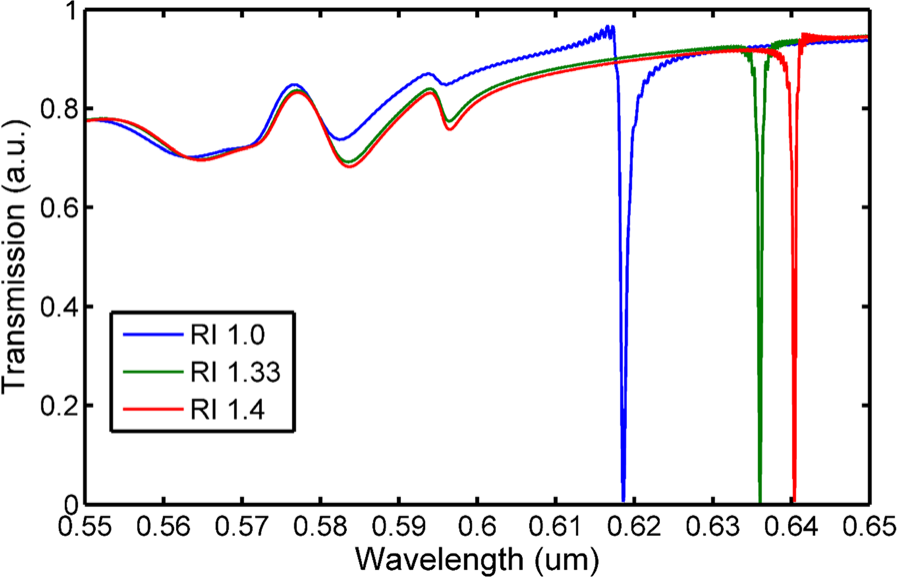
Simulation of transmission spectrum as reflective index of nanochannel variation

In order to test whether the analyte has flowed into the biosensor or not, a test method was designed and simulated. By modulating the refractive index in the nanofluidic channel, a series of PWV were obtained. If the PWV varies as the refractive index in the channel has changed, then it can be concluded that the analyte has flowed into the channel. This method can be regarded as a signal to test whether the fluid has successfully flowed into the nanochannels or not. When the channels are filled with analytes, the effective refractive index around the grating area will be changed since the refractive index of analyte is large than air. As shown in Fig. 4, the PWV is located at 618.6 nm when the material in the channel area is air (RI 1.0), and then the PWV shifts to 636 nm as the material modified to water (RI 1.33). The PWV can even shift to 640.4 nm as the refractive index rises to 1.4, which indicates the PWV will red shift when the refractive index of analyte in the nanosensors is gradually increased.

## Results and Discussion

### Bonding Effect Test

**Fig. 5:**
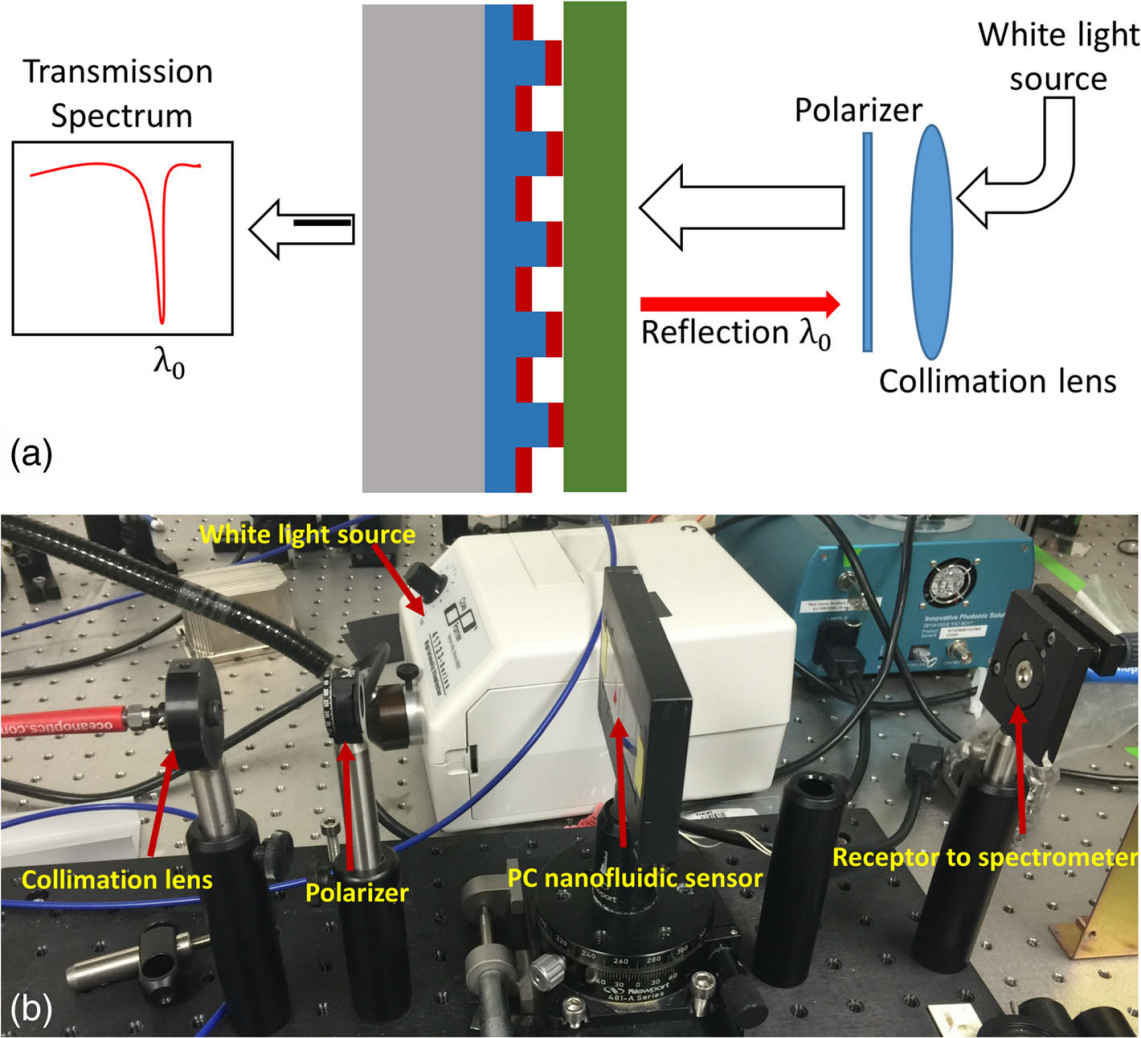
**a** Schematic of transmission setup. **b** The real measurement platform

**Fig. 6:**
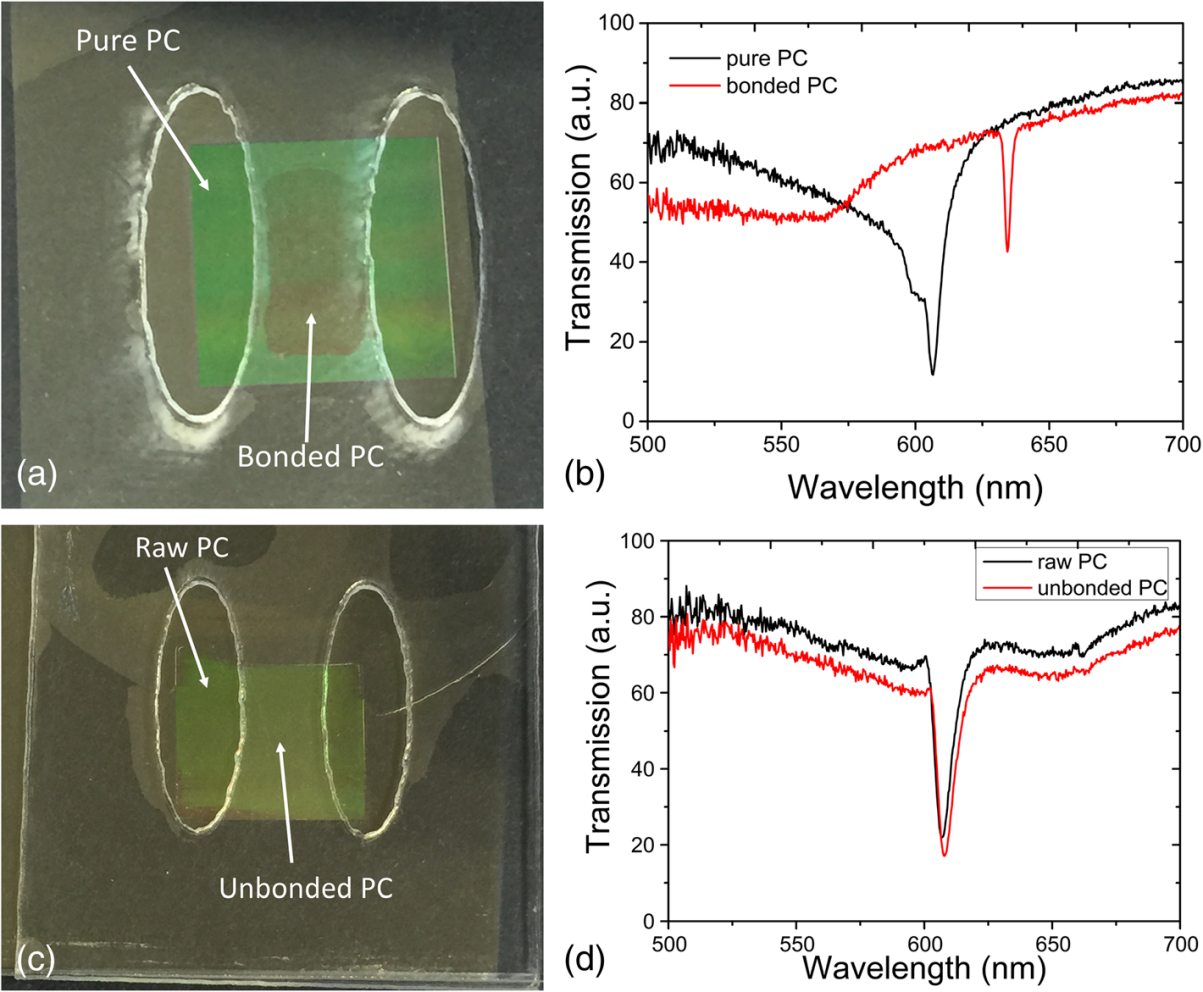
**a** Bonded PC-based biosensor. **b** Transmission spectrum of bonded PC. **c** Unbonded PC-based biosensor. **d** Transmission spectrum of unbonded PC

With the fabricated PC-based nanofluidic sensors, bonding effect experiments was used to test the bonding result. The transmission spectrum setup used for testing is shown in Fig. 5; a white light source is collimated by a collimation lens and TM polarized by a polarizer. Then, the collimated and TM polarized white light propagates into the photonic crystal-based nanofluidic structure. A specific wavelength light will resonate with periodic grating structure and reflect back, while the remaining wavelengths pass through the sensor. A spectrometer from OSA is used to collect the transmission spectrum. When the PC was bonded with the upper layer, a red shift was detected from the spectrometer. As the electrical field around the high refractive index grating area has changed, the effective refractive index of the PC is increased, which has conformed to the simulation result in Fig. 3b. When the PC was not bonded with the upper layer, the peak wavelength of the photonic crystal channel did not change. According to the simulation result, when the distance between the surface of the PC TiO2 layer and the bottom of the upper layer is larger than 50 nm, there will be no red shift of the PC peak wavelength value. The experiment tests match with the simulation result. Figure 6a was a well-bonded PC-based nanofluidic biosensor. From the related transmission spectrum in Fig. 6b, it indicated that the PWV of the raw PC is 606.6 nm and the PWV shifted to 634.8 nm when the PC was sealed by the top layer. However, in Fig. 6c, d, the PWV of pure PC and bonded PC remained on 610.4 nm. This is due to the reason that the bonding effect between the PC and top tape layer was not good, and there was a gap exist. From these two comparison experiments, it can be concluded that the PWV shift can be regarded as an indicator for the bonding result.

### Flow Ability Test

**Fig. 7:**
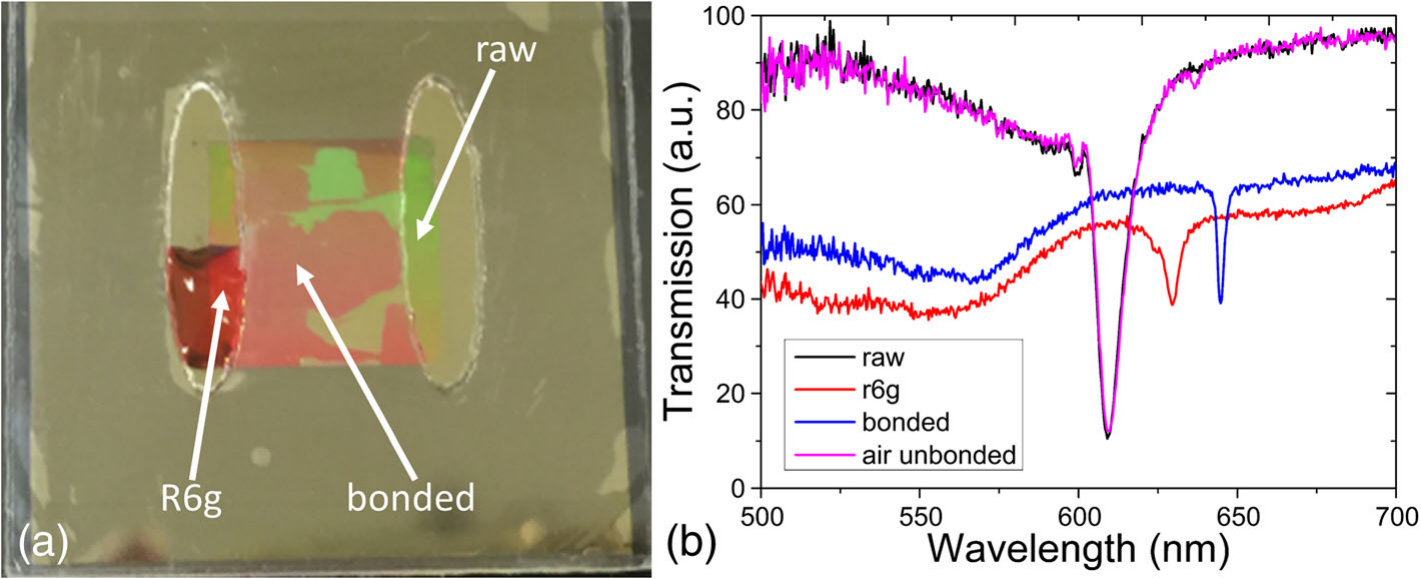
**a** Nanofluidic sensor with R6g flow through. **b** Related transmission spectrum of nanofluidic sensor with R6g

In order to test the flow ability of the nanofluidic channel, Rhodamine 6 g, which dropped on the inlet of the attached nanofluidic channel, was used as a liquid sample for testing. The analyte was gradually flowing into the nanofluidic sensor. A modified syringe was also used as a self-made pump to pump air from the outlet of the sensor, which can help the R6G flow to the outlet. As shown in Fig. 7a, the red R6g flowed from one end to the other side of the channel. As indicated from Fig. 7a, a portion of the bonded area was blocked, which may be due to the grating structure collapsed during the nanoreplica molding process or air particles contaminated and block the nanochannels. Another reason for the nonuniformity of the bonded area may remain on the method of bonding between PC and upper adhesive layer, and a portion of the adhesive layer may filled the channel area of the PC which block the fluidity of R6g as result. During the experiments, even a portion of bonded area may be blocked, and the nanofluidic sensor can still work normally. There are plenty of normal gratings that can be used as nanochannels, and the light source spot used to excite the sensor area is a spot with 2 mm diameter. In Fig. 7b, the raw photonic crystal PWV was acquired when the collimated and polarized white light source spot shine on the area of the raw PC area without any cover layer on the surface. The air-unbounded PWV was obtained by moving the source spot to the area of unbounded area. R6g PWV derived from the inlet area where R6g was dropped on the surface of the raw PC gratings. The bonded one was collected when white light source passed through the bonded area, where filled with R6g on the nanochannels. From Fig. 7b, it shows that the original PWV at 611.4 nm did not change when there bonded effect did not work, and then the PWV shifted to 629.5 nm as the raw grating surface was immersed with R6g liquid. When the R6g flowed into the bonded channel area, the PWV shifted significantly to 644.2 nm. It can be concluded that a portion of the nanochannels worked based on our fabrication method. This experiment had been repeated several times, and the results look promising. For example, there is another similar example of another nanoreplica molding sensor, as in Additional file 1: Figure S1. The peak wavelength value (PWV) of this raw PC is 606.3 nm, the PWV of bonded PC is 637.2 nm, and the PWV of R6g on PC is 635.3 nm. While for the contrast sensor on Fig. 7b, the PWV of pure PC is 611.4 nm, the PWV of bonded area is 644.2 nm and the PWV of R6g on PC is 629.6 nm. Both these sensors can be used for further application. When comparing these two sensors, the PWVs are different. The pure PC PWV of the first one is 606.3 nm while the second one is 611.4 nm. The main reason is fabrication parameter variation, such as grating depth of TiO2. During the TiO2 deposition process, the equipment fluctuation may cause the depth of deposited TiO2 shift from exactly 70 nm. While this photonic crystal structure is very sensitive with very high electrical field enhancement, the tiny various TiO2 depth can affect the position of PWV, as shown in Additional file 1: Figure S2, S3. Additional file 1: Figure S2 is the electrical field enhancement of the PC-based nanofluidic biosensor with the maximum E field enhancement factor larger than 90, and the red rectangular area is the channel area. Additional file 1: Figure S3 is a simulation of the peak wavelength value variation as the depth of TiO2 changes. From these two figures, it can be seen that each sensor may have different peak wavelength position due to the reason of parameter disturbance during fabrication. However, for a single sensor, the position of PWV variation does not affect the performance of the sensor. For label-free detection, the sensor is using peak wavelength shift as a signal for detection. For fluorescence enhancement experiments, the appropriate peak wavelength value can be obtained by tuning the incident angle of laser source.

### PCEF Experiment

**Fig. 8:**
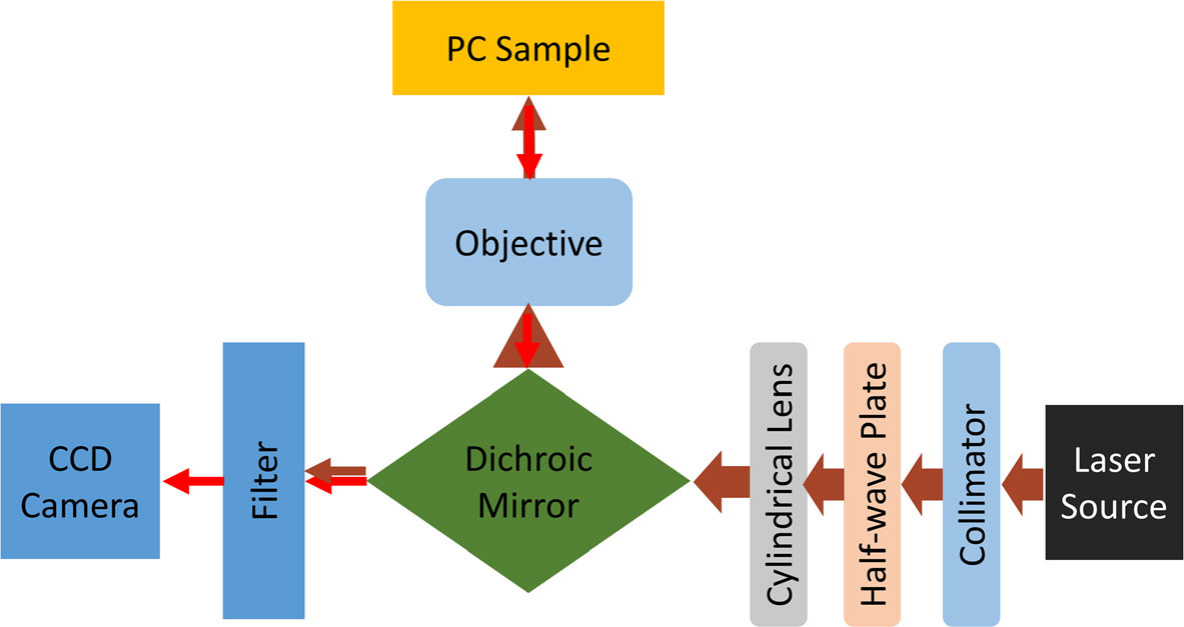
Schematic of PCEF platform. The laser source is collimated and polarized by collimator and half-wave plate respectively, then the cylindrical lens is used to focus the light into a line shape perpendicular to the direction of PC-based biosensor, and the emission fluorescence light is collected by an objective lens and constructed as an image after passing through a Dichroic mirror and laser source light filter

Fluorescence is an essential technique in the area of chemical, biological, and life science field due to its optical sensitivity and the extensive range of dye molecules. Especially in the low concentration or single molecule detection region, dye molecules that emit fluorescence signal are usually used as tag for these analytes. When the light source used to excite the fluorescence is located on the resonance wavelength range of PC, there will be a highlighted electrical field that exists to enhance the excitation of fluorescence. When the fluorescence signals emission from the dye molecules, the emission is also enhanced due to PC’s ability to redirect the direction of emission light. The emission and excitation enhancement, which are regarded as photonic crystal enhanced fluorescence (PCEF), can largely improve the intensity of the fluorescence signal and make it possible for low-concentration analyte detection. With the benefit of fluorescence enhancement, PC-based nanofluidic sensor can be used for fluorescence-based experiments. An initial PCEF experiment was settled in order to test the nanofluidic channels’ ability on the research area of fluorescence based biodetection. The PCEF was designed and configured by exciting the PC-based nanofluidic channel with a 70-mW, 637-nm GaAs laser source. As shown in Fig. 8, the laser source is collimated and TM polarized by collimator and half-wave plate respectively, then the cylindrical lens is used to focus the light into a line shape perpendicular to the direction of PC-based biosensor, and the emission fluorescence light is collected by an objective lens and constructed as an image after passing through a Dichroic mirror and laser source light filter. Also, the incident angle of the laser source can be tuned precisely, 0.01°, in order to find an incident angle that matches between the laser source wavelength and the resonance wavelength of the PC-based nanofluidic channels. With this illumination method, the intensity of light interacts with the sample can be largely improved. A line scanner camera was used to capture the scanning images while the laser beam source moves gradually along the detection area. Then, the sequence of line scanning images was saved and processed into a real 2D image for fluorescence light intensity analysis. When tuning the incident angle of the 637-nm laser source, the resonance wavelength of PC-based nanofluidic sensor will also shift.

**Fig. 9:**
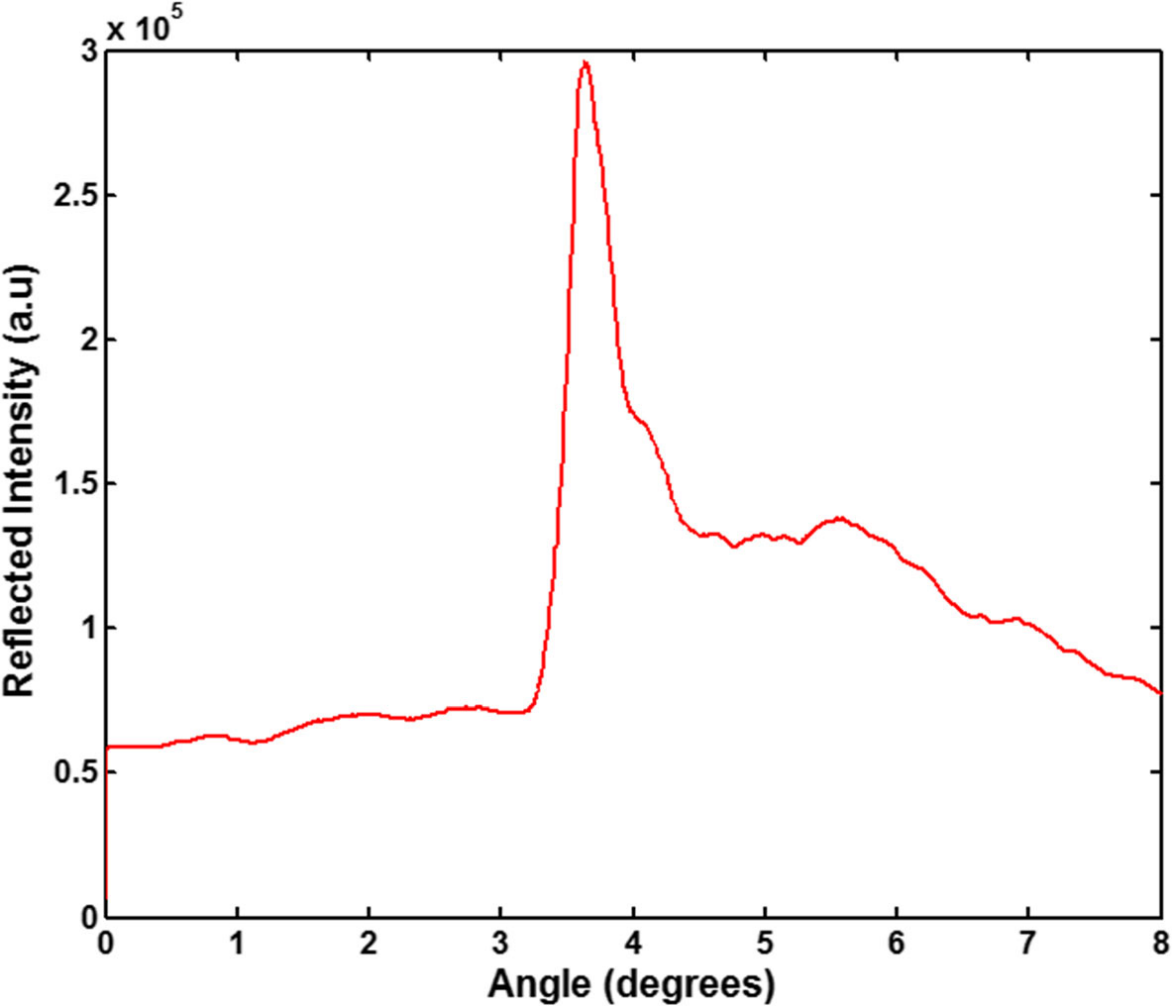
Reflection spectrum of PC-based biosensor with incident angles range from 0 to 8° excited by a 637-nm laser source

In order to figure out the incident angle of the laser source that can excite the PC resonance matches with each other, the PCEF line scanner setup which has a computer-controlled linear stage was used, where the incident angle of laser source can be tuned among a wide range from 0 to 20°. With this setup, a reflection spectrum of the nanofluidic sensor was obtained at fixed wavelength 637 nm while tuning the incident angle of laser source. As shown in Fig. 9, it indicated that the incident angle at which the resonance wavelength of nanofluidic sensor can match with the laser source is 3.64°, since it has the largest intensity among the spectrum.

**Fig. 10:**
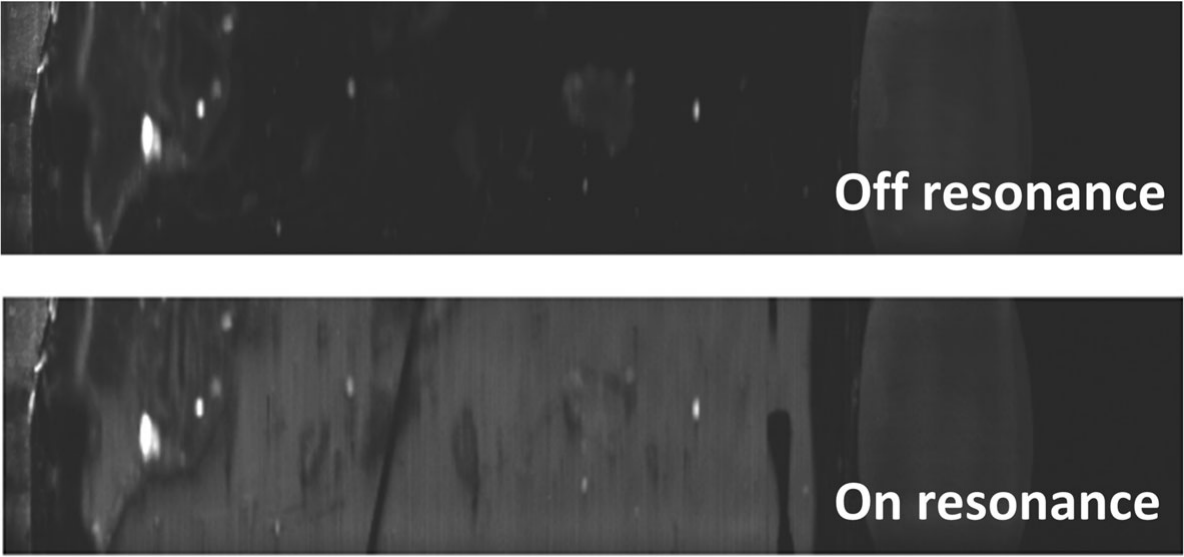
Line scanning fluorescence image of PC-based nanofluidic sensor: *on resonance* and *off resonance*

Alexa Fluor 635 from Thermal Fisher Scientific was used as fluorescence dye for PECF experiment. Then, the dried sample on the nanofluidic sensor was mounted on the PECF line scanner setup. According to the angle scanning result, the incident angle of the laser source was fixed at 3.64° as on resonance mode. Meanwhile, a contrast off resonance experiment was set at an incident angle of 0°. The fluorescence images of on and off resonance mode are shown in Fig. 10, and the on resonance one is much brighter than their off resonance one. The calculation of the fluorescence images were processed by ImageJ. An enhancement factor of 2.0 was obtained. The enhanced intensity of fluorescence indicates that the matched resonance PC-based sensor improves the fluorescence emission, which can be further used for fluorescence biological and chemical experiments.

## Conclusion

In this work, a novel PC-based nanofluidic biosensor structure was proposed, simulated, and fabricated. The nanoreplica molding PC biosensor with UV curable epoxy can be mass produced due to its low cost and replication characteristic. Peak resonance wavelength shift method was used to test the bonding effect of the PC and taped layer. Flow ability of the analyte was also analyzed based of the peak wavelength shift of the PC-based biosensor. R6g was used as the analyte for the related bonding effect and flow ability experiments. The results demonstrated the effectiveness of the peak resonance wavelength shift method. Also, the PC-based nanochannel biosensor was used for PCEF experiment. The incident angle of 3.64° was measured out for coupling the 637-nm laser source as the peak resonance wavelength of the PC biosensor. The PC biosensor was later used for the PCEF experiment with Alexa Fluor 635 fluorescence dye, and an enhancement factor of 2 was obtained. In conclusion, the descripted PC-based nanofluidic biosensor can be used for both label-free and enhanced fluorescence-based chemical, medical, and life science experiments. With the characteristic of nanoreplica molding, it can be mass produced and widely used in the future among related areas.

## Additional file

Additional file 1: Figure S1. Transmission spectrum of another R6g test with PC-based nanofluidic biosensor. **Figure S2.** Electrical field of PC-based nanofluidic biosensor. Period, 400 nm; TiO2 depth 70 nm; channel depth 120 nm. White line area, TiO2; red line area, channel area. **Figure S3.** TiO2 depth vs PC resonance wavelength shift. (DOCX 480 kb)

## Abbreviations

DI water: Deionized water
FDTD: Finite difference time domain
IPA: Isopropyl alcohol
PC: Photonic crystal
PCEF: Photonic crystal enhanced fluorescence
PWV: Peak wavelength value
TM: Transverse magnetic
UV: Ultraviolet
