# Supplementary material for: A Nanofluidic Biosensor Based on Nanoreplica Molding Photonic Crystal
Source: Nanoscale Res Lett. 2016 Sep 23;11:427. doi: 10.1186/s11671-016-1644-x (PMC5035292; doi:10.1186/s11671-016-1644-x)
Supplement: Additional file 1: Figure S1. — Transmission spectrum of another R6g test with PC-based nanofluidic biosensor. Figure S2. Electrical field of PC-based nanofluidic biosensor. Period, 400 nm; TiO2 depth 70 nm; channel depth 120 nm. White line area, TiO2; red line area, channel area. Figure S3. TiO2 depth vs PC resonance wavelength shift. (DOCX 480 kb) [file 11671_2016_1644_MOESM1_ESM.docx]

**Supplementary files:**


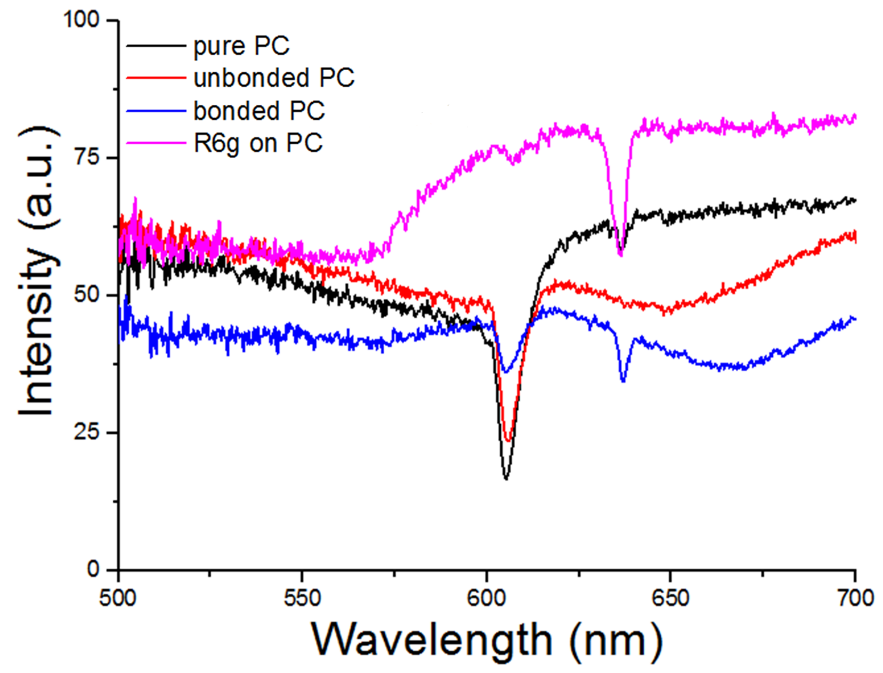


Fig. a Transmission spectrum of another R6g test with PC based nanofluidic biosensor.


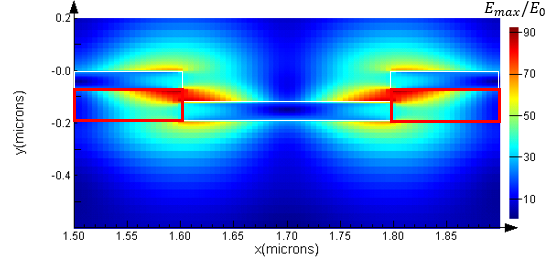


Fig. b Electrical field of PC based nanofluidic biosensor. Period, 400nm; TiO2 depth 70nm; Channel depth 120 nm. White line area, TiO2; Red line area, channel area.


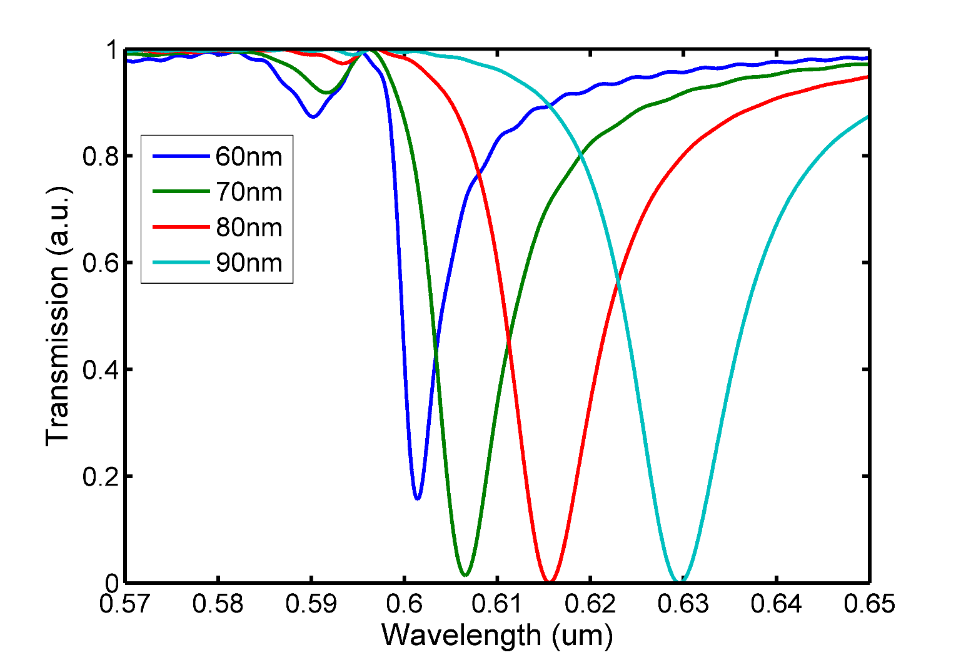


Fig. c TiO2 depth vs PC resonance wavelength shift.
